# Mental health of college students and associated factors in Hubei of China

**DOI:** 10.1371/journal.pone.0254183

**Published:** 2021-07-02

**Authors:** Xiaosheng Lei, Chaojie Liu, Heng Jiang

**Affiliations:** 1 School of Management, Hubei University of Chinese Medicine, Wuhan, P. R. China; 2 Department of Public Health, School of Psychology and Public Health, La Trobe University, Melbourne, Australia; 3 Centre for Alcohol Policy Research, School of Psychology and Public Health, La Trobe University, Melbourne, Australia; 4 Melbourne School of Population and Global Health, University of Melbourne, Melbourne, Australia; College of Medicine and Sagore Dutta Hospital, INDIA

## Abstract

**Background:**

Although many studies have analyzed mental health problems in college students in China, the associations of mental health with individual and family circumstances, academic performance, and social support were rarely discussed.

**Objectives:**

The study aimed to determine the mental health status and its associated factors among college students in China.

**Methods:**

An online survey was conducted on 300 students selected through a cluster sampling strategy in a university in Hubei, China, tapping into sociodemographic characteristics of the participants, their mental health status measured by the modified Symptom Checklist-90 (SCL-90), and social support measured by the Social Support Rating Scale. Multivariate linear regression models were established to identify predictors of the SCL-90 scores.

**Results:**

Mental health disorders were found in 8% of the respondents, with obsessive compulsive, interpersonal sensitivity and depression as the top three reported problems. Compared with the national population norms of university students in 2014, our study participants had higher scores in SCL-90. There were statistically significant difference in the *Global Severity index (GSI)* and all nine subscales scores (*p*<0.05). Poorer self-rated health, higher study pressure, and lower social support were significant predictors of higher SCL-90 scores (*p*<0.05) after adjustment for variations in other variables.

**Conclusions:**

College students in Hubei, China may experience a range of different mental health problems, which are associated with their individual, study, and social circumstances.

## 1. Introduction

Health is not simply an absence of physical illness, it also includes healthy personality and mental conditions. Mental health is an important element in overall health and wellbeing, which encompasses intellectual health, spiritual health and emotional health [1]. The World Psychiatric Association (WPA) proposes four standards of mental health: physical and mental coordination, adaptation to social environment, well-being, and bringing one’s ability into full play in work. According to the World Health Organization (WHO), mental health of college students involves a stable mood, coordinated interpersonal relation, objective self-judgement, and psychological adaption [2]. College students have to face more challenges by their own when they transition from adolescence to adulthood while leaving home to attend universities. This transition is often associated with an increase in depression, anxiety and stress [3–5]. Therefore, understanding how to properly treat the mental health problems plaguing college students is an important task in universities and the society.

In a survey conducted by the WHO, over 30% of college students from eight countries were estimated to have experienced mental distress [6], which seriously affected their study and daily lives, leading to role impairments and poor academic records [7–9]. An investigation in the US found that more than one in third college students were in depression and about 10% of the college students had considered suicide in the past year of 2008. The number of students with mental disorder appeared to be increasing in the last two decades [10]. A Chinese study in 2010 estimated that about 10%-30% of college students experienced mental health problems [11]. Common mental problems of college students include depression, compulsion, anxiety and interpersonal sensitivity [12–15]. Two recent systematic reviews found a prevalence of depression of 28.4% in Chinese college students, compared with 25% of postgraduate students suffering from mental illness [16, 17]. Therefore, understanding the prevalence and predictors associated with college students’ mental health could help to develop effective psychological interventions and reduce related adverse effects.

Previous studies found that socioeconomic factors such as gender and income are associated with mental health of college students [18, 19]. It has been reported that academic pressure and study loads, financial difficulty, away from home or unstable family, and campus bully can all increase the risk of mental health disorders [12, 13, 20, 21]. Since mental health of college students is shaped by both individual behaviors and social environments [22], research on mental health of college students should pay increasing attention to the unique challenges of university life as a preparation for future careers. A study in China revealed a link between mental health problems and poor interpersonal relationships in adolescents [15]. By contrast, a higher level of physical activities is associated with better mental well-being [23].

Recent studies show that social support has a critical impact on mental health [24]. College students in China face unprecedented academic pressures due to intensified job competitions. College students must pay tuition fees which presents a peculiar challenge to those from low-income households. Empirical evidence shows that the health impacts of environmental factors vary by socioeconomic backgrounds of individuals. The contextual characteristics often dictate how an individual responds to environmental challenges. Social support has been proved to be an effective tool to help individuals to mitigate some of the health risks of environmental challenges [25, 26]. Thus, we hypothesize that college students with a higher family socio-economic status and receiving stronger social support would experience fewer mental health problems in Hubei, China.

Although extensive studies have been conducted on mental health of college students in China, there is a paucity in the literature documenting a holistic view on the associations of mental health with individual and family circumstances, academic performance, and social support [27, 28]. This study explores the influencing factors from individuals, families, schools and society comprehensively. From a comprehensive perspective, this study aims to assess the mental health status of college students using the SCL-90 in comparison with the nationwide norms and determine its association with the socioeconomic characteristics and social support obtained by the students [29]. Findings of the study will provide a theoretical basis for psychological interventions in universities in China.

## 2. Methods

### 2.1 Study subjects

The study subjects were college students over 18 years old. An online survey was conducted in a university with over 20,000 enrolled students in Hubei of China from December 16, 2019 to January 19, 2020. The required sample size, calculated based on the effect size of 0.08 with 95% statistical power, is 293 participants. The study was approved by the ethics committee of Hubei University of Chinese Medicine.

### 2.2 Research design

A cluster sampling strategy was adopted to select study participants. Six out of the 12 schools with different disciplines in the university were randomly selected. The selected schools included Management, Literature and Law, Information Engineering, Physical Education and Health, Nursing, and Sanitary Inspection. The subjects were divided into medicine and non-medicine. One class group was randomly selected from each of six schools. All enrolled students in the identified class groups were invited to participate in the study and those who had accepted the invitation were further invited to participate in an online survey. The purpose and contents of the survey were explained to the participants before they provided informed consent. The weblink of the online Questionnaire were then sent to the students. Those who agreed to participate were asked to complete the online survey anonymously. Finally, a total of 302 questionnaires were returned and two were excluded in data analyses due to missing or illogical information. There were 300 valid questionnaires.

### 2.3 Measurements

The questionnaire contained two parts. Part one measured mental health using the modified Chinese version of SCL-90 instrument [30, 31]. The SCL-90 includes 90 items [32]. Respondents were asked to rate each item on a five-point Likert scale ranging from 1 = "none" to 5 = "severe". The scores were summed up (ranging from 90 to 450) to reflect severity of mental symptoms. Nine subscale average scores were also calculated, measuring *somatisation (SOM)*, *obsessive/compulsive (OBS)*, *interpersonal sensitivity (INT)*, *depression (DEP)*, *anxiety (ANX)*, *hostility (HOS)*, *phobia anxiety (PHOB)*, *paranoid ideation (PAR)*, *and psychoticism (PSY)*. The *Global Severity Index (GSI)* is equal to the average of all nine subscale scores. A higher score indicates a higher level of mental health problems. A subscale score of ≥3 was deemed clinical positive with symptoms [33]. The SCL-90 scale has been widely used in assessing mental health of college students in China. Exploratory factor and correlation analyses were adopted in many Chinese studies which demonstrated reliability and validity of SCL-90 Scale [34, 35]. In this study, high internal consistency and construct validity were demonstrated: Cronbach’s ɑ 0.975, KMO 0.945, and Bartlett’s test *p* < 0.001.

Part two measured factors that are potentially associated with mental health. The selection of the measurements was guided by the theory of social determinants of health (SDH). According to the SDH theory, mental health is not only a reflection of individual health behaviors, but also a product of the environments in which a person lives, studies, and works. In this study, we measured the university-specific SDH features, including *self-rated general health*, *study pressure*, *academic performance*, *physical exercises*, *and social support*. The variables were measured by participant’s subjective perception. *Self-rated health* was measured by a rating scale ranging from 1 (good health) to 3 (bad health). *Study pressure* was measured by self-perceived learning stress, with 1 indicating low, 2 indicating medium and 3 indicating high levels of stress. *Academic performance* was measured by a self-rating satisfaction scale, ranging from 1 (good) to 3 (bad). *Physical exercises* were measured by frequency, ranging from 1 (often doing exercises) to 3 (seldom doing exercises). *Social support* was measured by the 10-items social support rating scale (SSRS) developed by Xiao et al [36]. The SSRS measures the existence (1 = yes; 0 = no) of objective support (3 items), the degree (ranging from 1 to 4) of subjective support (4 items), and the utilisation of social support (3 items). The scores for each respondent were summed up, with ≤22 indicating low support, 23–44 indicating medium support and ≥45 indicating high support [37]. The SSRS scale is widely used in China, it has been proved with high reliability and validity by using t-test, correlation analysis, factor analysis and other statistical methods [38, 39]. Good internal consistency and structure validity were demonstrated in this study: Cronbach’s ɑ 0.756, KMO 0.710, Bartlett’s test *p*<0.001.

Control variables involved in this study included *gender*, *academic year*, *enrolled major*, *rural/urban location of households*, *and monthly family income*. Respondents of this study covered academic year one to year four. *Enrolled major* was divided into medicine and non-medicine. *Family monthly income per capita* was categorised into five groups: 1(<¥1500); 2 (¥1500–3000); 3 (¥3001–5000); 4 (¥5001–8000); 5 (>¥8000).

### 2.4 Statistical analysis

The SCL-90 total and its nine subscale scores were the major interest of this study. We tested differences in the SCL-90 scores among the respondents with various characteristics using student *t* tests or one-way analysis of variance (ANOVA) tests. Descriptive statistics, student-t test, ANOVA test and regression model were used to analyze the data.

Data were analysed using the Statistical Package for Social Science (SPSS) version 22.0. A *p* value of <0.05 was considered statistically significant.

## 3. Results

### 3.1 Characteristics of study participants

Of the 300 respondents, 15.3% were men and 84.7% were women. The majority came from year one (46.3%) and year two (40%). Rural students accounted for 65.7% of the respondents (Table 1).

**Table 1:** SCL-90 total scores of respondents by sociodemographic characteristics.

Characteristics	n (%)	SCL-90 Score (Mean±SD)	t or F value	*p*	Paired comparison of SCL-90 Score (LSD test)
**Gender**					
Male	46 (15.3)	148.76 ± 44.73	-1.377	0.170	
Female	254 (84.7)	158.40 ± 43.49			
**Residency**					
Urban	103 (34.3)	152.95 ± 46.01	-1.137	0.257	
Rural	197 (65.7)	158.99 ± 42.48			
**Enrolled major**					
Medicine	56 (18.7)	164.45 ± 52.86	1.430	0.154	
Non-medicine	244 (81.3)	155.19 ± 41.30			
**Grade**					
1	139 (46.3)	157.22 ± 41.16	0.754	0.471	
2	120 (40.0)	154.19 ± 45.41			
3, 4	41 (13.7)	163.88 ± 47.47			
**Physical exercise**					
Often	36 (12.0)	133.64 ± 27.01	6.481	0.002**	Often < Occasionally
Occasionally	191 (63.7)	158.50 ± 44.08			Often < Seldom
Seldom	73 (24.3)	164.26 ± 46.16			
**Self-rated health**					
Good	153 (51.0)	144.74 ± 37.28	29.241	0.000***	Good < General < Bad
General	141 (47.0)	165.88 ± 42.25			
Bad	6 (2.0)	257.00 ± 58.08			
**Study pressure**					
Low	15 (5.0)	125.73 ± 18.61	20.888	0.000***	Low < Medium < High
Medium	255 (85.0)	153.79 ± 39.43			
High	30 (10.0)	199.13 ± 59.28			
**Academic performance**					
Good	10 (3.3)	146.70 ± 50.90	7.173	0.001**	
General	252 (84.0)	153.65 ± 41.08			General < Poor
Poor	38 (12.7)	181.32 ± 51.71			
**Family monthly income per capita (Chinese Yuan)**					
< 1500	83 (27.7)	160.86 ± 42.83	0.944	0.439	
1500–3000	168 (56.0)	157.40 ± 44.10			
3001–5000	27 (9.0)	142.56 ± 32.78			
5001–8000	17 (5.7)	158.12 ± 61.17			
>8000	5 (1.6)	149.00 ± 21.93			
**Social support**					
High (> 44)	24 (8.0)	132.42 ± 32.49	11.466	0.000***	High < Medium < Low
Medium (23–44)	271 (90.3)	157.75 ± 42.72			
Low (< 23)	5 (1.7)	229.60 ± 59.54			
**Total**	300	156.52 ± 43.74			

Note

**: *p*< 0.01

***: **p**< 0.001.

### 3.2 Mental health status of respondents

The respondents had a mean score of 156.92 (SD = 43.74) in the SCL-90 total. The mean of the *Global Severity index* (GSI) was 1.74 (SD = 0.19). About 8% reported positive symptoms in at least one of the nine subscales (subscale score ≥3).

The top three reported problems were obsessive, interpersonal sensitivity and depression (Table 2). Compared with the new nationwide norms for college students in 2014 (N = 4456) [30], our respondents had higher scores in SCL-90. There were statistically significant difference in GSI and all nine subscales scores (*p*<0.05).

**Table 2:** Comparison of SCL-90 subscale scores between study participants and nationwide norms of college students (x ± s).

Subscale	The present study (N = 300)	National Norms (N = 4456)	t	*P*
Somatization	1.52±0.46	1.36±0.46	6.098	0.000***
Obsessive compulsive	2.10±0.54	1.77±0.63	10.504	0.000***
Interpersonal sensitivity	1.97±0.63	1.60±0.60	10.264	0.000***
Depression	1.84±0.57	1.52±0.58	9.616	0.000***
Anxiety	1.69±0.57	1.49±0.57	6.177	0.000***
Hostility	1.65±0.57	1.46±0.55	5.721	0.000***
Phobia	1.56±0.58	1.36±0.51	6.090	0.000***
Paranoia	1.65±0.49	1.46±0.53	6.857	0.000***
Psychoticism	1.66±0.56	1.44±0.52	6.921	0.000***
Global Severity Index	1.74±0.19	1.50±0.49	8.680	0.000***

Note

*** P< 0.001.

A subscale average score of ≥3 was deemed clinical positive with symptoms [34]. 6.7% of the participants had an average score of ≥3 in OBS, indicating obvious symptoms of obsessive compulsive. 7.7% of the participants had positive symptoms in interpersonal sensitivity. 3% of the participants were positive for depression.

### 3.3 Factors associated with mental health

No gender, academic year, residency and income differences were found in the SCL-90 total scores. Lower SCL-90 total scores (*p*<0.05) were associated with good self-rated health and more frequent physical exercises. The respondents with a higher study pressure and poorer academic performance had higher SCL-90 scores (*p*<0.01). The respondents reported an average score of 35.83 (SD = 6.063) in social support: 19.22±3.627 for subjective support, 8.96±2.513 for objective support, and 7.65±1.712 for utilisation of support, respectively. Higher levels of social support were associated with lower SCL-90 scores (*p*<0.05, Table 1). Collinearity diagnosis tests revealed no significant collinearity between the independent variables included in the multivariate linear regression analyses, with variance inflation factor (VIF) ranging from 1.094 to 1.194. The multivariate regression model showed that self-rated health, study pressure and social support were significant predictors (*p*<0.05) of the SCL-90 total score after adjustment of variations in other variables (Table 3).

**Table 3:** Factors associated with the SCL-90 total score: Results of linear regression analyses.

Variables	Unadjusted		Adjusted *β*	t	*p*
	*β*	Standard Error			
**Constant**	2.311	0.289		8.003	< 0.001
**Self-rated health** (1 = Good, 2 = General, 3 = Bad)	0.232	0.048	0.257	4.86	< 0.001
**Study pressure** (1 = Low, 2 = medium, 3 = High)	0.27	0.069	0.214	3.905	< 0.001
**Academic performance** (1 = Good, 2 = General, 3 = Poor)	0.058	0.067	0.047	0.868	0.386
**Physical exercise** (1 = Often, 2 = Occasionally, 3 = Seldom)	0.038	0.044	0.046	0.861	0.39
**Social support** (1 = High, 2 = Medium, 3 = Low)	0.017	0.004	0.207	3.744	< 0.001

Further modelling on the nine SCL-90 subscales confirmed that self-rated health, study pressure, and social support were independent predictors of the subscale scores, except for phobia (Table 4). Self-rated health was the biggest predictor, followed by study pressure and social support.

**Table 4:** Results of multivariate linear regression modeling on SCL-90 subscale scores.

SCL-90 Subscale	Adjusted β coefficient (Standard Error)				
	Self-rated health	Study pressure	Academic record	Physical exercise	Social support
Somatization	0.223(0.047)***	0.167(0.068)**	0.112 (0.067)*	0.078 (0.043)	0.134(0.061)*
Compulsion	0.194 (0.056)***	0.137 (0.080)*	0.096 (0.079)	0.150 (0.051)**	0.119 (0.075)*
Interpersonal relationship	0.205 (0.064)***	0.222 (0.092)***	0.056 (0.090)	0.062 (0.059)	0.146 (0.083)**
Depression	0.219(0.057)***	0.217 (0.082)***	0.057 (0.080)	0.079 (0.052)	0.183 (0.074)***
Anxiety	0.257 (0.057)***	0.268 (0.082)***	-0.015 (0.080)	0.036 (0.052)	0.139 (0.074)*
Hostility	0.216 (0.059)***	0.224 (0.084)***	0.080 (0.082)	0.005 (0.054)	0.121 (0.076)*
Phobia	0.248 (0.060)***	0.194 (0.086)**	-0.006 (0.920)	0.024 (0.055)	0.095 (0.078)
Paranoia	0.225 (0.050)***	0.213 (0.072)***	0.041 (0.071)	0.014 (0.046)	0.152 (0.065)**
Psychoticism	0.252 (0.056)***	0.212 (0.080)***	0.056 (0.079)	-0.007 (0.051)	0.183 (0.073)***

Note

* P< 0.05

** P< 0.01

*** P< 0.001.

## 4. Discussion

### 4.1 Mental health of college students

This study revealed a prevalence of 8% mental health disorders in the study participants. Mental health problems are common among college students [40]. Our study identified obsessive, interpersonal sensitivity and depression as top three mental health problems in the study participants, which is consistent with findings of previous studies [20]. Compared with the nationwide norms of college students in 2014, the study participants had higher scores in GSI and all nine subscales.

Since the 21st century, mental health education of college students in China has been greatly developed. China has the largest number of college students in the world. With the rapid socioeconomic development in China, college students are facing more social competition and pressures in study and employment, which might result in the increase of psychological problems. Moreover, the study period was just before the period of coronavirus lockdown in Hubei. There might be some potential impact of the early surge of COVID on students’ mental health in this study. Additionally, most of the current college students are the only child in the family, who may not be good at dealing with interpersonal and social problems. Those might be the reasons for the deviation from the national scores.

### 4.2 Factors associated with mental health of college students

Our study found no differences in the SCL-90 total score across groups of participants with different gender, academic year, enrolled major, residency and family income. These results are not completely consistent with findings of previous studies. Some previous studies found gender, academic year and household income were associated with mental health of college students [18]. Female students account for the majority of participants in this study. Along with social economic development, gender inequality in university education has been gradually reduced in China over the last three decades. Both male and female students are encouraged to develop knowledge, talents and skills. The mental health status of college students is likely to evolve with these changes, which may offer some explanations of the inconsistent findings. In addition, a small number of senior college students participated in this survey, which might explain the difference of academic year on mental health was not significant. The increasing availability of university subsidies, student loans and academic scholarships for college students in recent years may reduce the potential association between family income and mental health.

College students face great challenges in academic studies because they have the potential to determine life-long career of the students. Our study shows that higher study pressure and poorer academic performance are associated with higher SCL-90 scores. These results are consistent with findings of previous studies [4]. Study difficulties are likely to be linked with increased study pressure and poor academic performance. The overall health of students may suffer as a consequence as revealed in this study and others.

Physical exercises and social support were found to be positively associated with mental health in this study. Previous studies have proved that regular exercises can help reduce stress and improve mental health [41–43]. Building a strong social network such as making more close friends and participating in community activities can help college students to manage study and life challenges and difficulties. Family support remains to be essential. Although college students are away from families, they are not independent adults yet.

College students are the future of a country. Although knowledge acquittance is important, it should not be achieved at the cost of mental health. Universities should develop a care culture and environment that supports the life adjustment of college students, promotes cultural and sports activities, and facilitates the expansion of social networks. Meanwhile, mental health education and psychological counselling services should be strengthened. These include hotlines offering timely help to these in urgent needs. Early detection and effective management of mental health problems can effectively reduce serious mental health disorders.

### 4.3 Limitation

This study has some limitations. The study sample was drawn from one university. Since mental health is highly context dependent, the results can not be extrapolated to the entire university sector. In addition, this is a cross-sectional study which could not reflect the development of mental health problems in students over university life. The mental health problems were self-rated by students which may have a recall bias. Further comparative studies are needed to better understand the underlying mechanisms of mental health problems in college students and their long term impacts on the career development of the students, so as to provide policy or psychological intervention and reduce mental problems among university students in China.

## 5. Conclusions

Mental health problems are common in college students in China, which can be reflected on different aspects. Obsessive compulsion, interpersonal sensitivity and depression are top three reported problems in the university studied. Self-rated health, study pressure, and social support are significant predictors of mental health in college students.
